# Influence of Molecular Orbitals on Magnetic Properties of FeO_2_H_*x*_

**DOI:** 10.3390/molecules25092211

**Published:** 2020-05-08

**Authors:** Alexey O. Shorikov, Sergey L. Skornyakov, Vladimir I. Anisimov, Sergey V. Streltsov, Alexander I. Poteryaev

**Affiliations:** 1 M.N. Miheev Institute of Metal Physics of Ural Branch of Russian Academy of Sciences, 620108 Ekaterinburg, Russia; shorikov@imp.uran.ru (A.O.S.); skornyakov@imp.uran.ru (S.L.S.); via@imp.uran.ru (V.I.A.); streltsov@imp.uran.ru (S.V.S.); 2 Theoretical Physics and Applied Mathematics Department, Ural Federal University, Mira St. 19, 620002 Ekaterinburg, Russia; 3 Skolkovo Institute of Science and Technology, 3 Nobel Street, 143026 Moscow, Russia

**Keywords:** iron dioxide, DMFT, DFT+DMFT, magnetism, high pressure

## Abstract

Recent discoveries of various novel iron oxides and hydrides, which become stable at very high pressure and temperature, are extremely important for geoscience. In this paper, we report the results of an investigation on the electronic structure and magnetic properties of the hydride FeO${}_{2}$H${}_{x}$, using density functional theory plus dynamical mean-field theory (DFT+DMFT) calculations. An increase in the hydrogen concentration resulted in the destruction of dimeric oxygen pairs and, hence, a specific band structure of FeO${}_{2}$ with strongly hybridized Fe-$t_{2g}$-O-$p_{z}$ anti-bonding molecular orbitals, which led to a metallic state with the Fe ions at nearly 3+. Increasing the H concentration resulted in effective mass enhancement growth which indicated an increase in the magnetic moment localization. The calculated static momentum-resolved spin susceptibility demonstrated that an incommensurate antiferromagnetic (AFM) order was expected for FeO${}_{2}$, whereas strong ferromagnetic (FM) fluctuations were observed for FeO${}_{2}$H.

## 1. Introduction

In the study of the formation of molecular orbitals, organic compounds are prominent. Classes of materials where molecular orbitals can be found are certainly not exclusive. There are plenty of inorganic systems where the physical properties can be strongly affected by the formation of molecular orbitals (see, e.g., [1,2,3,4]); however, it may be surprising to find any molecular orbitals in the minerals of the lower part of Earth’s mantle, which experiences huge pressure and incredibly large temperatures. Nevertheless, molecular orbitals are a key component of FeO${}_{2}$ material, which is now believed to be one of the main constituents of the Earth’s mantle [5,6,7,8,9,10].

The crystal structure of FeO${}_{2}$ is the same as the well-known pyrite, FeS${}_{2}$ [5,11]. Iron is in the oxygen octahedra; thus they share their corners, and some of the oxygens form dimer-like structures. These dimeric O atoms and formed molecular orbitals were shown to play a crucial role and strongly affect the valence state of Fe, and the electronic structure and magnetic properties of FeO${}_{2}$[12]. In particular, in contrast to the sister compound, FeS${}_{2}$, iron dioxide was predicted to be a “bad metal” with a pseudogap at the Fermi level, a paramagnet with unusual—for transition metal oxides—temperature behaviour of the magnetic susceptibility (increasing with temperature) and unexpected valencies of the Fe and dimeric O–O pair, which are, however, intensively debated at present [10,12,13].

**Figure 1:**
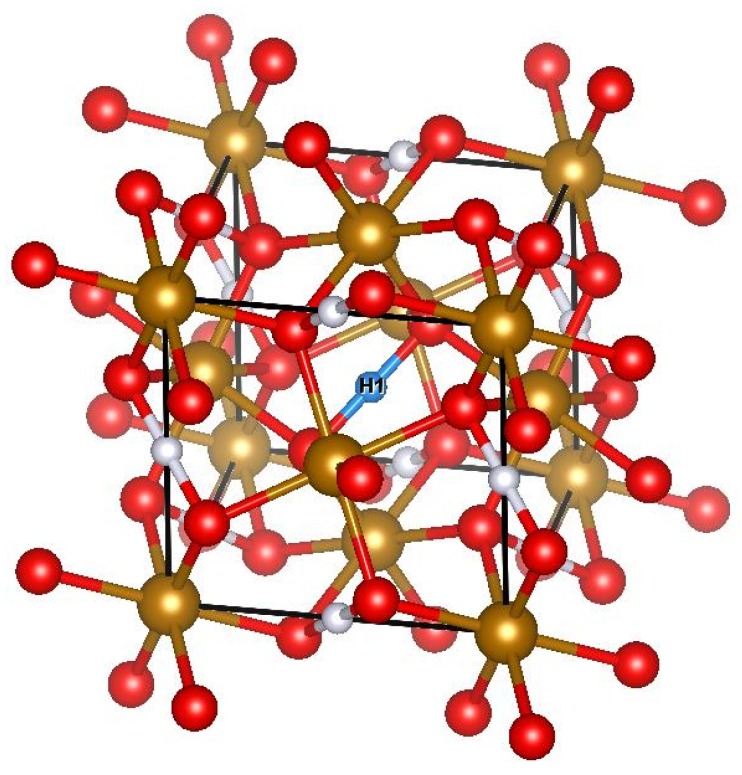
(Color online) Crystal structure of FeO_2_H. The space group is $Pa\bar{3}$. Iron and oxygen ions are shown by bronze and red colors, respectively. Central hydrogen is denoted H1 and shown by cyan, while the rest of H are shown by light grey. All hydrogens are equivalent in this crystal structure.

Destruction of the dimeric O–O and corresponding molecular orbitals by a proton (which sits exactly in the middle of the O${}_{2}$ bond in FeO${}_{2}$H, see Figure 1) strongly modifies its physical properties. In contrast to pure FeO${}_{2}$, stoichiometric FeO${}_{2}$H was shown to behave like a normal weakly correlated system with localized electrons and magnetic susceptibility following the Curie–Weiss law [14]. However, experimental high-pressure studies demonstrated that in real materials, which can be synthesized in a lab, one typically obtains FeO${}_{2}$H${}_{x}$ with *x* close to 0.5 [7]. Unfortunately, not much is known regarding the physical properties of such a nonstoichiometric situation. The present paper aims to fill this gap in the understanding of the electronic and magnetic structure of nonstoichiometric FeO${}_{2}$H${}_{x}$.

## 2. Results

### 2.1. Structural Properties

For a relaxation of the crystal structure, we used the pseudo-potential VASP package [15] in a framework of generalized gradient approximation after Perdew, Burke and Ernzerhof (GGA-PBE) [16]. The crystal shape, volume, and atomic position were relaxed at the pressure of 119 GPa [8]. The energy cutoff was 1000 eV, and a 7 × 7 × 7 Γ-centered k-mesh was utilized for all calculations.

Figure 1 shows the crystal structure of FeO_2_H, which has a $Pa\bar{3}$ space group. The iron ions (bronze color) are in 4a Wyckoff positions and they are surrounded by an octahedra of oxygen (red). The oxygen ions are in 8c Wyckoff positions with x=0.35225, the hydrogens are in 4b positions. The site symmetry of all ions contains a threefold rotational axis. The octahedra are corner shared with a node connecting three different octahedra.

All Fe-O distances are the same, $d_{Fe-O}$ = 1.79 Å. The octahedron is slightly squeezed along the direction perpendicular to the face resulting in O-Fe-O angles equal to 97.48 degrees and 82.52 degrees. The hydrogens (blue and light grey) are located in a large inter-octahedra space, which exists in this geometry. One should note here that all H are equivalent but colored differently for the sake of better visibility and later discussion.

The crystal structures of FeO_2_H_*x*_ can be obtained by a consecutive removing of H. This results in the linear decrease of the unit cell volume shown (see Table 1). A partial removing of H (because *x* < 1) leads to symmetry lowering of the space group with two kinds of Fe ions and, hence, the surrounding octahedra. The volumes of the octahedra of both types grow with the increase of the H concentration. The octahedra’s volumes are very close to each other in numbers. The Fe-O distance in the pure FeO${}_{2}$  is $d_{Fe-O}$ = 1.75 Å  and the smallest O–O distance in the octahedron is 2.33 Å.

At the same time, the distance between two oxygen atoms, which belongs to different octahedra is 15% smaller, $d_{O-O}$ = 1.99 Å. This short O–O distance is responsible for the formation of the bonding state and reduction of the oxygen valency [12]. Figure 1 can be used to visualize the positions of these two atoms. In this picture, this short distance can be viewed as one with the H1 legend. The inserted hydrogen increases the distances between all such type of pairs of oxygens (see rightmost column of the Table 1). For example, at the smallest calculated concentration of hydrogen, FeO_2_H_0.25_, the sole H is located at the center (H1) and the corresponding distance is $d_{O-H-O}$ = 2.19 Å, while the other distances (can be regarded as light grey) are $d_{O-O}$ = 2.05 Å. Therefore, the hydrogen insertion rotates the octahedra in such a way that increases the inter-octahedra space.

### 2.2. GGA-PBE Band Structure

**Figure 2:**
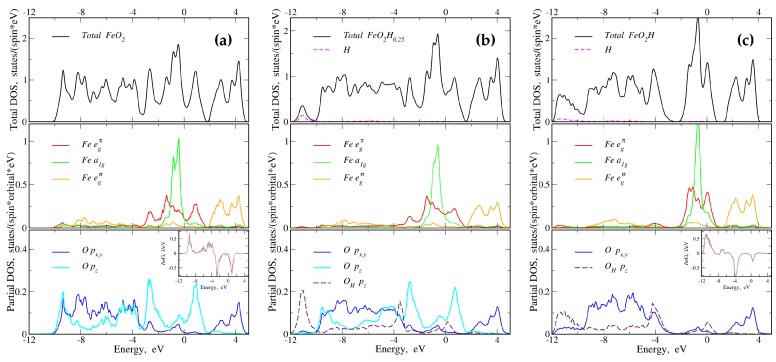
(Color online) The total and partial density of states for FeO_2_H_*x*_. (**a**) Pure FeO${}_{2}$, (**b**) FeO_2_H_0.25_, and (**c**) FeO_2_H. Top panels: the total density of states (DOS) is in black. The H states are shown by the dashed magenta color. Middle panels: the orbital resolved partial DOS of iron. The red, green, and orange colors represent the $e_{g}^{\pi }$, $a_{1g}$ and $e_{g}^{\sigma }$ states, respectively. Lower panels: the orbital resolved partial DOS of oxygen. The blue color shows the double degenerate $p_{x}$ and $p_{y}$ states, and the cyan color is for $p_{z}$ orbital. The insets in the bottom panels show the imaginary part for the off-diagonal Green function in the pair of the nearest oxygen atoms (closest to H1).

The total and partial density of states (DOS) for FeO${}_{2}$  are presented in Figure 2a. It is typical for many oxide materials to form completely occupied O 2*p* bands and transition metal 3*d* bands above [17,18,19]. The Fermi level is located on the slope of the DOS, which is predominantly of Fe *d* character. The partial DOSes for all atoms are shown in the local coordinate frame with local *z* axis for Fe pointing to the octahedron face [20] and coinciding with a cell diagonal. The local *z* axis of the O atom that is the nearest to H1 is also along the cell diagonal and looks to H1.

The local coordinate systems of the remaining atoms can be obtained by symmetry operations. Hereafter the local coordinate frame notations will be used. In the octahedral environment, the *d* band of iron is split onto $t_{2g}$ and $e_{g}^{\sigma }$ sub-bands. The later (orange color in the middle panel) is directed to the nearest oxygen and it is strongly hybridized with the degenerate O $p_{x,y}$ states (blue color in the lower panel, local coordinate system notations) and it is located from 2 eV to 4.7 eV. The $t_{2g}$ band is further split onto $a_{1g}$ ($d_{z^{2}}$ orbital in the local coordinate system) and $e_{g}^{\pi }$ (green and red colors in the middle panel, respectively). The former is almost four times narrower, and that is due to the hybridization of the $e_{g}^{\pi }$ states with the O $p_{z}$ (cyan color in the lower panel). This hybridization leads to a formation of the bonding peak at about −2.6 eV and the antibonding peak at 0.9 eV.

Figure 2b shows the total and partial DOSes for FeO_2_H_0.25_. The addition of one hydrogen to the unit cell results in the symmetry lowering with two crystallographically different Fe and O ions. One additional electron in the system leads to a small shift of the Fermi level to a higher energy (in Figure 2b,c it looks visually as a shift of the Fe $e_{g}^{\sigma }$ and upper band-edge of $e_{g}^{\pi }$ partial DOSes to lower energies). The partial DOSes for iron ions are almost identical and are presented in averaged way. The shape of the $e_{g}^{\pi }$ partial DOS and its bandwidth are almost the same as in pure FeO${}_{2}$. This is due to the volume of the octahedra, and thus the Fe-O distances, which is only slightly increased and most of the O $p_{z}$ orbitals (cyan color) are still participating in the bonding with the Fe $e_{g}^{\pi }$ states (red color).

At the same time, the $p_{z}$ orbital of the closest to H oxygen (denoted as O${}_{H}$ $p_{z}$; dashed maroon in the bottom panel) points directly to the hydrogen. This particular spacial orientation leads to a destruction of the O–O bonding state and the O${}_{H}$ $p_{z}$ orbital does not participate in $p_{z}$-$e_{g}^{\pi }$ bonding anymore. Instead, it is hybridized with the hydrogen (dashed magenta in the top panel) and this shifts the O${}_{H}$ $p_{z}$ band down to −11 eV. One should emphasize here that this hybrid band is not a new band that appears below the O *p* manifold, but it becomes “isolated” due to large splitting.

In the completely hydrogenated FeO_2_H  all dimeric O–O states are destroyed and the O $p_{z}$ orbitals do not hybridize with the Fe $e_{g}^{\pi }$ states. The destruction of the dimeric state can be seen in the insets of Figure 2a,c, where the imaginary part for the off-diagonal Green function is shown. This off-diagonal Green function (corresponding to well-known in chemistry the crystal orbital overlap population curve [21]) is defined as:

(1) $$G\left(\varepsilon \right)=\sum_{k}^{BZ}\sum_{n}\frac{C_{in}^{+}\left(k\right)C_{jn}\left(k\right)}{\varepsilon -e_{n}\left(k\right)},$$

where the sums are performed over the Brillouin zone and all Kohn–Sham eigenvalues, $e_{n}\left(k\right)$. $C_{i(j)n}\left(k\right)$ are eigenvectors, which correspond to $p_{z}$ orbitals of different oxygen atoms located at opposite sides of H1 atom (see Figure 1). One can clearly see that when the hydrogen atoms is inserted between two oxygen atoms, the shape of the Green function becomes asymmetric, indicating the loss of the bonding–antibonding state. This results in a strong reduction of the $e_{g}^{\pi }$ bandwidth from 4.6 eV in pure FeO${}_{2}$  to 2.5 eV in FeO_2_H  (see Figure 2c). The Fe $a_{1g}$ and $e_{g}^{\sigma }$ and O *p* bands (excluding the subband at −11 eV) are about ten percent decreased in width, which is caused by the volume expansion. Therefore, there are two effects due to the addition of the hydrogen to the system. The major is a breaking of the O–O bonds with a strong reconstruction in the partial DOS within Fe *d* manifold. The secondary effect is the bandwidth reduction.

### 2.3. The Density Functional Theory plus Dynamical Mean-Field Theory (DFT+DMFT) Results

In spite of a quite successful description of the broad band metallic systems, the density functional theory fails often in a case of open shell transition metal compounds, and it malfunctions completely above the magnetic critical temperature, where the paramagnetic regime with local magnetic moments sets in. The DFT+U approach [22] can conceptually describe systems with a partial filling of shells and long range magnetic ordering; however, this approach fails again at describing paramagnetic metallic states. Even more, when treating correlation effects on a static mean-field level, the DFT+U approach often overestimates the tendency of system to be magnetic.

In this view, the use of a more accurate theory that treats the correlation effects dynamically is more preferable. Therefore, studying of the magnetic properties of the compounds of interest the combination of the density functional theory and the dynamical mean field theory were used [23,24]. The former introduces the material specific aspects of the problem describing delocalized states, while the later is able to treat strong on-site Coulomb correlations in the paramagnetic regime properly. Additionally, the DFT+U approach is a static limit of the DFT+DMFT method [25].

For the DFT+DMFT calculations, the AMULET package was used [26] with the continuous time quantum Monte Carlo method for a solution of impurity problem [27]. The non-magnetic DFT bands were projected onto the O 2p and Fe 3d states [28]. The projected bands span the energy interval from −12 to 5 eV and coincided completely with the Kohn–Sham bands. The Fe 3d orbitals were regarded as correlated with the values of the screened Coulomb interaction and Hund’s exchange were 6 eV and 0.89 eV, respectively [14]. During the DFT+DMFT calculations, we did not restrict the system to a particular magnetic order (if it is not stated otherwise).

The correlation effects were relatively weak and reflect themselves mainly via renormalization of the non-interacting band structure near the Fermi level. The effective mass enhancement, $m^{*}/m$ is equal to 1.19 for $e_{g}^{\sigma }$, and 1.38 and 1.33 for $a_{1g}$ and $e_{g}^{\pi }$ orbitals, respectively. The change of the H concentration leads to a smooth increase of the mass renormalization factor (see Table 2). For both types of iron atoms $m^{*}/m$ values grow up and become 1.24, 1.51, and 1.57 for the $e_{g}^{\sigma }$, $a_{1g}$, and $e_{g}^{\pi }$ states in completely hydrogenated FeO_2_H, respectively.

In paramagnets, a magnetic moment quickly fluctuates, which leads to $\langle m_{z}\rangle =0\mu_{B}$, where $m_{z}=\sum_{i}\left(n_{i}^{↑}-n_{i}^{↓}\right)$. At the same time, the instant squared magnetic moment, $\langle m_{z}^{2}\rangle$, is not zero and it can be directly evaluated in the quantum Monte Carlo, which is used in the DMFT approach. For iron dioxide the instant squared magnetic moment was found to be 2.45 $\mu_{B}^{2}$, which is slightly larger than 1 $\mu_{B}^{2}$ for the low spin $3d^{5}$ configuration. The value of the instant squared magnetic moment decrease slightly with increasing the hydrogen content and become $\langle m_{z}^{2}\rangle$=2.25 $\mu_{B}^{2}$ for FeO${}_{2}$H. This can be explained simply by increasing the *d*-shell occupancy from 6.2 electrons in FeO${}_{2}$ to 6.3 electrons in FeO${}_{2}$H, which leads to a smaller magnetic polarization.

For all hydrogen compositions, the analysis of the atomic configurations shows that FeO_2_H_*x*_ stays in the low spin state configuration. The hole doping results in a larger value of $\langle m_{z}^{2}\rangle$, and is connected with the smaller number of *d* electrons. At the same time, the Fermi level is shifted to the big $a_{1g}$ peak leading to a large value of mass re-normalization, $m^{*}/m$=2.23, for this orbital. Therefore, the hole doping transfers the compound to the correlated regime more efficiently even without destroying the O–O molecular orbitals as in FeO_2_H.

**Figure 3:**
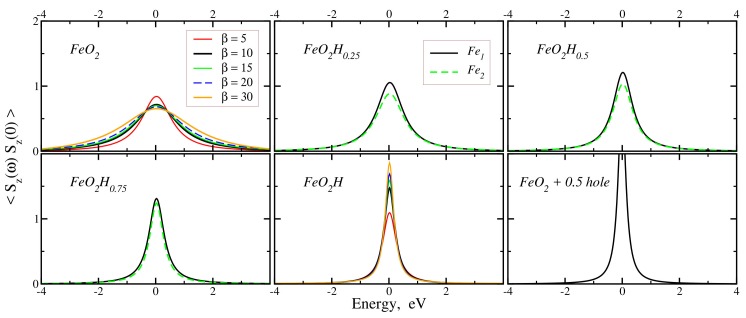
(Color online) FeO_2_H_*x*_ spin–spin correlation functions for different values of concentration, *x*, at β = 10 eV${}^{-1}$. Left-top panel: the temperature dependence for pure FeO${}_{2}$. Middle-top, right-top, left-bottom: local spin–spin correlation functions for the corresponding concentrations. Middle-bottom: the temperature dependence for FeO_2_H. Right-bottom: the spin–spin correlation function for FeO${}_{2}$+ 0.5 hole ($\chi_{loc}\left(0\right)\approx 2.83$).

The local spin–spin correlation functions, $\chi_{loc}\left(\omega \right)=\langle S_{z}\left(\omega \right)S_{z}\left(0\right)\rangle$, can be successfully used as a measure of the local moment localization degree [29,30]. Its width is inversely proportional to the lifetime of the local magnetic moment and the value at zero frequency is a quarter of the instant squared magnetic moment, $\chi_{loc}\left(0\right)=\langle m_{z}^{2}\rangle /4$. Figure 3 shows the $\chi_{loc}\left(\omega \right)$ for various concentrations of hydrogen. One can clearly see that the electron doping results in the increase of the instant squared magnetic moment by a factor of two, approximately. The different iron atoms display very similar behavior for the local spin–spin correlation function, which is consistent with the values of quasi-particle mass enhancements (see Table 2).

The full width at half maximum (FWHM) is decreased with hydrogen doping that implies a stronger localization of the magnetic moment in completely doped FeO_2_H. At the same time, the value of FWHM in FeO_2_H is about 0.5 eV, which is much larger than in α-Fe or Fe${}_{2}$O${}_{3}$, where the local magnetic moments are well established and developed [14,29], hence concluding that the magnetic moment is not localized. The hole doping results in a shift of the chemical potential from the deep DOS to the slope of the peak of the $a_{1g}$ character (see Figure 2a). This increases strongly the correlation effects as the system gets away from the completely filled $t_{2g}$ subshell with a low spin state and results in increase of the magnetic moment (right-bottom panel in Figure 3).

It is interesting to analyze the temperature dependence of the correlator, $\langle S_{z}\left(\omega \right)S_{z}\left(0\right)\rangle$, in pure FeO${}_{2}$ and FeO_2_H (see Figure 3). In completely hydrogenated FeO_2_H, where all O–O bonds are destroyed and Fe does not participate in the Fe $e_{g}^{\pi }$ O $p_{z}$ bonding, the $\langle m_{z}^{2}\rangle$ decreases with increasing temperature [17]. At the same time, in FeO${}_{2}$ the $\langle m_{z}^{2}\rangle$ increases with the increasing temperature. In the later case, the temperature becomes large enough to compete with hybridization effects and to destroy the Fe $e_{g}^{\pi }$-O $p_{z}$ bonding, and hence, guiding the system into the regime of paramagnetic Fe ions with a larger magnetic moment.

We calculated the static momentum-resolved spin susceptibility χ(q,T) employing the particle-hole bubble approximation [31]:

(2) $$\begin{array}{c} \chi \left(q,T\right)=-\frac{1}{\beta }\mathrm{Tr}\sum_{\begin{array}{c} k,i\omega_{n} \end{array}}\hat{G}\left(q,i\omega_{n}\right)\hat{G}\left(k+q,i\omega_{n}\right). \end{array}$$

Here $\hat{G}\left(k,i\omega_{n}\right)={\left[\left(i\omega_{n}+\mu \right)\hat{I}-{\hat{H}}_{\mathrm{DFT}}\left(k\right)+\hat{\Sigma }\left(i\omega_{n}\right)\right]}^{-1}$ is the lattice Green’s function, $i\omega_{n}=\left(2n+1\right)\pi /\beta$ are the fermionic Matsubara frequencies, $\beta =1/k_{B}T$ is an inverse temperature, μ is the chemical potential, $\hat{I}$ is the identity operator, and $\hat{\Sigma }\left(i\omega_{n}\right)$ is the local self-energy. The ${\hat{H}}_{\mathrm{DFT}}\left(k\right)$ denotes the effective Hamiltonian computed by projection onto a set of Wannier functions with symmetry of the O *p* and Fe *d* states.

**Figure 4:**
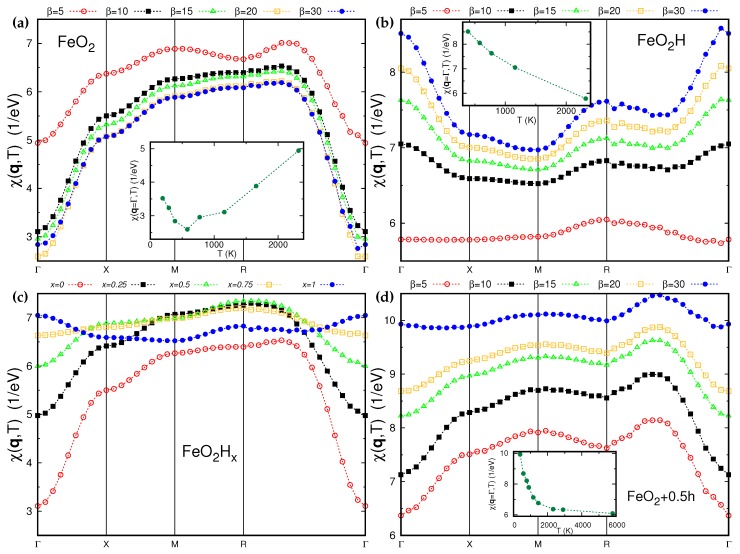
(Color online) The momentum-resolved spin susceptibility, χ(q,T), computed according to Equation (2) along the high symmetry directions of the Brillouin zone for different inverse temperatures. The insets show the temperature dependence of the corresponding susceptibility at the Γ point. (**a**) The temperature dependence of χ(q,T) for pure FeO${}_{2}$, (**b**) the temperature dependence of χ(q,T) for completely hydrogenated FeO_2_H, (**c**) χ(q,β=10) as a function of hydrogen concentration in FeO_2_H_*x*_, (**d**) the temperature dependence of χ(q,T) for 0.5 hole doped FeO${}_{2}$. For the color coding, please see the figure’s legend.

The momentum-resolved spin susceptibilities along the high symmetry directions in the Brillouin zone for different hydrogen concentrations and temperatures are presented in Figure 4. Figure 4a shows the temperature dependence of χ(q,T) for pure FeO${}_{2}$. The maximum of the function is located close to the middle of the R−Γ direction. One should note that at the highest temperature, β=5 eV${}^{-1}$, the second maximum begins to develop at the M point, thus indicating a different competing antiferromagnetic order. The non-trivial temperature dependence of susceptibility at the Γ point is presented in the inset. It is associated with peculiarities of the band structure of FeO${}_{2}$. At low temperatures, the Fermi level lies at the slope of the DOS of $a_{1g}$ character predominantly. The temperature increasing results in shifting the Fermi energy to deep in the DOS and to the Fe $e_{g}^{\pi }$-O $p_{z}$ antibonding states at 1 eV (see Figure 2a) [12].

The concentration dependence of the susceptibility is shown in Figure 4c for β = 10 eV${}^{-1}$. The maximum of χ(q,T) goes from the middle of the R−Γ direction towards the R point with the increasing hydrogen concentration. It becomes a global maximum for FeO_2_H_0.75_. For the complete hydrogenated case, i.e., for FeO${}_{2}$H, the R point is a local maximum, while the global one is at the Γ point indicating the favor of the ferromagnetic spacial correlations. One may naively explain this fact by the double exchange-like mechanism (see, e.g., [32,33]). As was discussed above, the hydrogenation is in some way analogous to the doping. One may expect to stabilize ferromagnetism in the system with localized spins doping it by metallic carriers.

The momentum-resolved spin susceptibility for FeO_2_H, shown in Figure 4b, is very temperature sensitive, which is manifested by change of the maximum of q point from Γ to R with the temperature increasing. However, iron moments order neither ferromagnetically nor antiferromagnetically (AFM type I) at the end-member compositions, FeO${}_{2}$ and FeO_2_H. FeO${}_{2}$ stays paramagnetic down to 190 K (we checked the FM and AFM-I orders). Even lower temperatures can be achieved using the Hamiltonian with a smaller dimension, which includes only the Fe $t_{2g}$ and O 2p states. This choice of correlated impurity orbitals gives the same spectral functions in the vicinity of the Fermi level and very similar uniform magnetic susceptibility as in full Hamiltonian, but it is less time consuming.

In this case, we were able to go down to 60 K and again FeO${}_{2}$ does not produce order at these temperatures. The Fe 3*d* bandwidth is quite large and comparable with the screened Coulomb interaction; thus, we might expect a large superexchange interaction between the Fe ions in the case of localized spins. In the case of metallic FeO${}_{2}$ or FeO_2_H we might expect that the exchange interaction not only between the nearest but also between te next nearest neighbors will be substantial. In the case of the face centered cubic lattice formed by Fe ions, these competing interactions may strongly suppress a long range magnetic order (see e.g., [34]).

Figure 4d shows the static spin susceptibility for FeO${}_{2}$ doped by 0.5 of the hole. In this situation, the maximum of χ(q,T) is again close to the middle of the R−Γ direction for all temperatures under consideration. This can be understood as a consequence of the presence of the O–O bonds and equivalence of the crystal structure used for these calculations. At the same time, χ(q=Γ,T), shown in the inset of Figure 4d, is qualitatively different from the pure FeO${}_{2}$. This is again connected with the band structure features. In the hole doped case, the Fermi level is shifted to the narrow and sharp $a_{1g}$ peak, which leads to the development of the larger local magnetic moment and stronger mass re-normalization (see Table 2).

## 3. Conclusions

We carried out the DFT and DFT+DMFT calculations of the electronic and magnetic properties of the stoichiometric FeO${}_{2}$ and FeO${}_{2}$H${}_{x}$ series for a number of hydrogen concentrations. The analysis of the DFT DOSes revealed that hydrogen doping destroyed the dimeric O–O pair and resulted in a complete reorganization of the band structure near the Fermi level: the broad peak corresponding to the Fe $e_{g}^{\pi }$-O $p_{z}$ hybridization band vanished and the whole width of the $e_{g}^{\pi }$ band decreased almost twice. This led to an increase of the effective mass enhancement, $m^{*}/m$, from 1.4 for FeO${}_{2}$ to 1.6 for FeO${}_{2}$H, decreasing the full width at half maximum of the corresponding local spin–spin correlation functions and increasing of the instant squared magnetic moment by a factor of two, approximately. This indicates that increasing the hydrogen concentration in FeO${}_{2}$H${}_{x}$ led to the growth of the localization degree of the magnetic moment. The temperature dependence of the momentum-resolved spin susceptibility showed that the χ(q,T) maximum shifted from the middle of the R−Γ direction for FeO${}_{2}$ to the R point for FeO${}_{2}$H${}_{0.75}$ and then to the Γ point for FeO${}_{2}$H, which corresponded to the transition from the AFM to FM fluctuations.

## Figures and Tables

**Table 1:** The structural properties of FeO${}_{2}$H${}_{x}$ for various hydrogen concentrations, *x* (left column). The second column is the unit cell volume. The third column is the volume of octahedra (the values in parenthesis correspond to second type of Fe). The right column is the distance between the nearest oxygen atoms (via hydrogen in parenthesis).

*x*	$V_{\mathrm{cell}}$, Å${}^{3}$	$V_{\mathrm{oct}}$, Å${}^{3}$	$d_{O-(H)-O}$, Å
0.0	76.243	6.961	1.993
0.25	78.010	7.101 (7.019)	2.053 (2.186)
0.5	79.786	7.169 (7.114)	2.118 (2.206)
0.75	81.451	7.266 (7.243)	2.175 (2.221)
1.0	83.057	(7.395)	(2.233)

**Table 2:** Orbitally-resolved enhancement of the band mass, $m^{*}/m$, and the instant squared magnetic moment, $\langle m_{z}^{2}\rangle$, for two types of iron atoms in FeO${}_{2}$H${}_{x}$ for different orbitals of the *d* shell as obtained by DFT+DMFT at β=10 eV${}^{-1}$ (data for the second type of Fe are in parenthesis). The last row shows the result for FeO${}_{2}$  doped with 0.5 of hole.

		$m^{*}/m$		$\langle m_{z}^{2}\rangle$
x	$e_{g}^{\pi }$	$a_{1g}$	$e_{g}^{\sigma }$	
0	1.38	1.33	1.18	2.45
0.25	1.46 (1.43)	1.42 (1.40)	1.20 (1.20)	2.66 (2.41)
0.5	1.46 (1.44)	1.42 (1.44)	1.20 (1.21)	2.61 (2.30)
0.75	1.48 (1.56)	1.48 (1.48)	1.22 (1.23)	2.45 (2.16)
1.0	(1.57)	(1.51)	(1.24)	(2.25)
0 +0.5*h*	1.50	2.23	1.19	3.47
